# Influence of probiotics on the periodontium, the oral microbiota and the immune response during orthodontic treatment in adolescent and adult patients (ProMB Trial): study protocol for a prospective, double-blind, controlled, randomized clinical trial

**DOI:** 10.1186/s12903-022-02180-8

**Published:** 2022-04-27

**Authors:** Corinna L. Seidel, Roman G. Gerlach, Matthias Weider, Theresa Wölfel, Vincent Schwarz, Armin Ströbel, Helga Schmetzer, Christian Bogdan, Lina Gölz

**Affiliations:** 1 Department of Orthodontics and Orofacial Orthopedics, Universitätsklinikum Erlangen and Friedrich-Alexander Universität (FAU) Erlangen-Nürnberg, Glückstr. 11, 91054 Erlangen, Germany; 2 Mikrobiologisches Institut-Klinische Mikrobiologie, Immunologie und Hygiene, Universitätsklinikum Erlangen and Friedrich-Alexander Universität (FAU) Erlangen-Nürnberg, Wasserturmstraße 3/5, 91054 Erlangen, Germany; 3 Center for Clinical Studies (CCS), Universitätsklinikum Erlangen and Friedrich-Alexander Universität (FAU) Erlangen-Nürnberg, Krankenhausstr. 12, 91054 Erlangen, Germany; 4 Med III, University Hospital of Munich, Workgroup: Immune Modulation, Marchioninistraße 15, 81377 Munich, Germany; 5 Medical Immunology Campus Erlangen, Friedrich-Alexander Universität (FAU) Erlangen-Nürnberg, 91054 Erlangen, Germany

**Keywords:** Probiotics, *Lactobacillus reuteri*, *Limosilactobacillus reuteri*, Oral microbiota, Cytokines, Inflammation, Orthodontic treatment, Oral health, Prevention, Study protocol

## Abstract

**Background:**

Orthodontic treatment with fixed appliances is often necessary to correct malocclusions in adolescence or adulthood. However, oral hygiene is complicated by appliances, and prior studies indicate that they may trigger oral inflammation and dysbiosis of the oral microbiota, especially during the first 3 months after insertion, and, thus, may present a risk for inflammatory oral diseases. In recent periodontal therapeutic studies, probiotics have been applied to improve clinical parameters and reduce local inflammation. However, limited knowledge exists concerning the effects of probiotics in orthodontics. Therefore, the aim of our study is to evaluate the impact of probiotics during orthodontic treatment.

**Methods:**

This study is a monocentric, randomized, double blind, controlled clinical study to investigate the effectiveness of daily adjuvant use of *Limosilactobacillus reuteri* (Prodentis®-lozenges, DSM 17938, ATCC PTA 5289) versus control lozenges during the first three months of orthodontic treatment with fixed appliances. Following power analysis, a total of 34 adolescent patients (age 12–17) and 34 adult patients (18 years and older) undergoing orthodontic treatment at the University Hospital Erlangen will be assigned into 2 parallel groups using a randomization plan for each age group. The primary outcome measure is the change of the gingival index after 4 weeks. Secondary outcomes include the probing pocket depth, the modified plaque index, the composition of the oral microbiota, the local cytokine expression and—only for adults—serum cytokine levels and the frequencies of cells of the innate and adaptive immune system in peripheral blood.

**Discussion:**

Preventive strategies in everyday orthodontic practice include oral hygiene instructions and regular dental cleaning. Innovative methods, like adjuvant use of oral probiotics, are missing. The aim of this study is to analyse, whether probiotics can improve clinical parameters, reduce inflammation and prevent dysbiosis of the oral microbiota during orthodontic treatment. If successful, this study will provide the basis for a new strategy of prophylaxis of oral dysbiosis-related diseases during treatment with fixed appliances.

***Trial registration*:**

This trial is registered at ClinicalTrials.gov in two parts under the number NCT04598633 (Adolescents, registration date 10/22/2020), and NCT04606186 (Adults, registration date 10/28/2020).

T0: Base line; Study time point before the insertion of the fixed appliance.

T1: Study time point ~ 1 week after insertion of the fixed appliance/ start of lozenge intake.

T2: Study time point ~ 4 weeks after insertion of the fixed appliance/ start of lozenge intake.

T3: Study time point ~ 12 weeks after insertion of the fixed appliance/ start of lozenge intake = last day of lozenge intake.

T4: Study time point ~ 24 weeks after insertion of the fixed appliance = 24 weeks after start of lozenge intake = 12 weeks after end of lozenge intake.

T_mem:_ Memory T cells.

T_reg_: Regulatory T cells.

Th_1_: Helper T cells: Th_1_ cells.

Th_2_: Helper T cells: Th_2_ cells.

Th_17_: Helper T cells: Th_17_ cells.

TNF: Tumor-necrosis-factor.

U: Sublingual area.

w: Week(s).

WHO: World Health Organisation.

WRT: Wilcoxon rank-sum test.

WSL: White spot lesions.

## Background

Orthodontic treatment with fixed appliances is frequently required to correct malocclusions in different age groups. The need of treatment can be determined by objective criteria and different indexes [1], e.g., the Index of Orthodontic Treatment Need (IOTN) [2]. Recently, it was shown that malocclusions have a negative impact on oral health-related quality of life (OHRQOL) [3], predominantly in adolescents [4]. However, it is known that fixed braces complicate oral hygiene and require more intense tooth cleaning, since biofilm accumulation increases, while self-cleaning through the tongue and saliva flow are reduced [5], thereby enhancing the risk for gingival and periodontal inflammation [3]. Periodontal inflammation was paralleled by the release of pro-inflammatory cytokines, acute phase reactions [6, 7], the occurrence of diabetes [8] and atherosclerosis [9], as well as by the presence of certain pathogenic bacterial species. *Porphyromonas (P.) gingivalis* was associated with atherosclerosis (T_reg_/Th_17_ imbalance) [10–13], Alzheimer's disease [14], rheumatoid arthritis [7, 15] and diabetes [16], and both *P. gingivalis* and *Fusobacterium (F.) nucleatum* were linked to adverse pregnancy outcomes [7, 17, 18]. Considering local inflammatory side effects, it is known that forces exerted during orthodontic treatment lead to stress in the periodontal apparatus of teeth and induce an aseptic pseudo-inflammatory response with the release of cytokines, e.g., tumor necrosis factor (TNF), Interferon-γ (IFN-γ) and Interleukin (IL)-1, IL-2, IL-3, IL-6 and IL-8 [19, 20]. On the other hand, the immune response against bacterial pathogens in periodontal disease is also mediated by humoral mediators [21] such as the aforementioned proinflammatory cytokines (IL-1, IL-2, IL-6, IL-8, TNF, IFN-γ) [22–27], which bears the risk for progressive destruction of the periodontium [28] and root resorptions [29, 30] requiring. Thus, strategies to inhibit the change from aseptic pseudo-inflammatory response to dysbiotic inflammatory disease during orthodontic treatment are necessary.

Bonded brackets, wires, ligatures and elastics create plaque retention niches [5] and were shown to change the oral bacterial composition [31, 32]. The oral cavity is colonized by over 700 bacterial species [33, 34] and presented three microbial metaniches [(1) plaque (P)—gingival crevicular fluid (GCF), (2) saliva (S)—tongue (T)—hard palate (HP) and (3) cheek (C)—sublingual area (U)] as well as similar immunological niche-/metaniche characteristics in young adults with oral health [34]. Considering the longitudinal changes, subgingival pathogens, e.g., *Aggregatibacter [A.] actinomycetemcomitans*, *P. gingivalis*, *Prevotella* [*P.*] *intermedia* and *Tannerella [T.] forsythia*, increased directly after the insertion of fixed braces for 3 months and returned to initial levels 6 months after appliance insertion [32]. An increase in periodontal pathogenic germs at the beginning of orthodontic treatment can lead to a flare-up of periodontal inflammation [28] especially in adults with age-related changes of oral microbiota [35] and the elevated incidence of periodontal diseases [36].

Probiotics are defined as preparations that contain living microorganisms in quantities sufficient to reduce dysbiosis and inflammation, e.g., in the gut [37]^38^ and in the oral cavity [39] via induction of immunomodulatory effects (production of anti-inflammatory, inhibition pro-inflammatory cytokines) [40–44]. Considering periodontal therapy, the therapeutic intake of *Limosilactobacillus [L.] reuteri* correlated with improved clinical parameters in patients at risk for gingivitis and periodontal disease: pregnant women and navy sailors [45, 46]. Since orthodontic patients represent another risk group, the use of probiotics might become a promising preventive strategy [47]. However, so far no positive effects were seen with regard to white-spot-lesions [48] or gingival inflammation [49–51] when probiotics were given for a short period (2–4 weeks) during the onset of orthodontic treatment [49–51]. It was concluded [47] that probiotics should be used earlier, i.e. before the development of gingival inflammation or periodontal problems, and it was recommended to analyse a longer intake of lozenges and the combination of more than one probiotic strain along with a higher number of follow-up appointments [47]. Hence, a clinical trial is needed to investigate the effects of the combined defined probiotic strains [47] (*L. reuteri* DSM 17938, *L. reuteri* ATCC PTA 5289 provided by BioGaia AB, Stockholm, Sweden) during orthodontic treatment with a longer daily intake of lozenges [47] starting at the time of insertion of the fixed appliance [47], considering different oral metaniches [52] and to evaluate possible systemic effects in elderly patients at risk for periodontal disease [7]. The overarching goal of the planned randomized clinical trial is to define preventive strategies for daily practice.

## Methods/design

### Objectives

The trial aims to analyse probiotics as a possible preventive treatment approach during orthodontic treatment with fixed appliances. The primary objective is to study the influence of adjuvant intake of test versus control lozenges during the first 3 months of the orthodontic treatment of adolescent and adult patients with fixed appliances on the gingival index.The secondary objective is the investigation of the influence of adjuvant intake of test versus control lozenges during the first 3 months of the orthodontic treatment of adult patients with fixed appliances on clinical parameters, local cytokine expression and the composition of the oral microbiota at different time points during the first 6 months of treatment. Moreover, the secondary objectives for the adult group also include the analysis of the influence of adjuvant intake of test versus control lozenges on systemic parameters and inflammation at different time points during the first 3 months of treatment.

The null hypothesis is that there are no significant differences between patients with adjuvant intake of probiotics (*L. reuteri* Prodentis®) during the first 3 months of the orthodontic treatment with fixed appliances and patients with adjuvant intake of control lozenges concerning the following criteria: gingival Index (GI), modified plaque index (MPI), periodontal probing depth (PPD), local cytokine expression and composition of the oral microbiota (in one representative of each of the three oral metaniches: P (representing P—GCF), T (representing S—T—HP) and C (representing C—U) [52]), serum cytokine levels and frequencies of innate and adaptive immune cells in peripheral blood of adult patients.

### Trial design

**Fig. 1:**
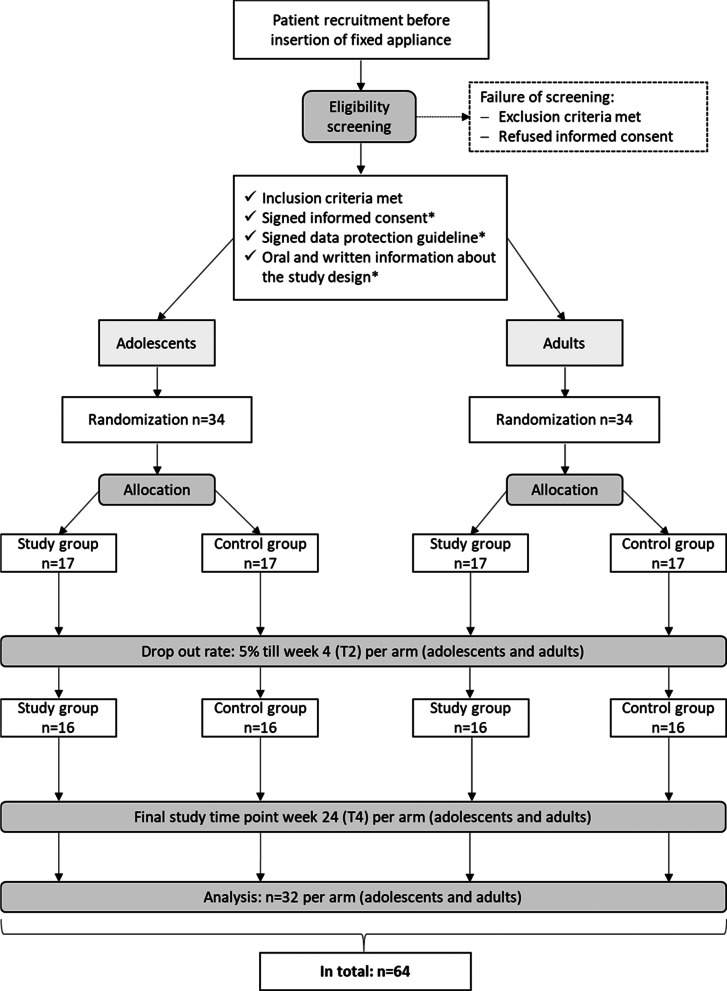
Trial protocol flow chart. n = number; *for the patient and/or legal guardian

The trial is designed as a monocentric, randomized, double-blind (observer and patient), controlled, two-armed clinical study (Fig. 1). The trial consists of two parts: a two-armed clinical study considering adolescents and a two-armed clinical study considering adults. The primary endpoint is the change of the GI from baseline to four weeks after insertion of fixed appliance and start of lozenge intake. Randomization will be conducted twice (for adults and adolescents) using the Maximal Procedure [53] with a 1:1 allocation. The sample size calculation is based on the change of the gingival index from baseline till week 4 and is explained in detail below (Sample size {14}). The estimated sample size is 16 per arm and 32 study participants in total with an estimated drop-out rate of 5%. Hence, the recruitment and allocation include 34 patients. The study period for each study participant is from baseline till week 24.

### Study setting

Since the study was designed as a monocentric clinical trial, the entire recruitment of patients will be performed at the Department of Orthodontics and Orofacial Orthopedics at the Friedrich-Alexander-Universität (FAU) Erlangen-Nürnberg, Germany. Currently, the Department of Orthodontics and Orofacial Orthopedics consists of one professor (the head of the department (L.G.)), two senior physicians (including C.L.S.), two specialists for orthodontics and four dentists in further education for the specialization as orthodontists. Orthodontic treatment of patients takes place from Monday till Friday and is performed by all five specialists for orthodontics and the four dentists, which are supervised by the professor, the senior physicians and the specialists. The study design was explained to all of the afore-mentioned colleagues as well as the inclusion/ exclusion list and all of the information material for patients (the declaration of consent, information about data protection and general information material). Patient recruitment will be performed by the study leader (C.L.S.) as well as orthodontists and dentists working at the department.

### Eligibilitey criteria

The inclusion criteria will consider the following:

- Adolescents from age 12 to 17 with fixed appliances undergoing orthodontic treatment
- Adults (18 years and older) with fixed appliances undergoing orthodontic treatment.
- Signed informed consent and declaration of consent by the patient and the parent or legal guardian for adolescent patients

The exclusion criteria will consider the following:

- Person affected by active periodontal disease (classification of periodontitis and disease severity will be performed in accordance to the new classification scheme by Caton et al. [54]) or that have undergone periodontal therapy within the last six months before insertion of fixed appliance.
- Person with systemic or metabolic diseases that can cause or modulate gingivitis (e.g., diabetes, active juvenile rheumatoid arthritis, leukemia, cancer) or could possibly influence the oral microbiome (e.g., chemotherapy, radiation therapy).
- Person affected by obesity: body mass index (BMI) > 30 kg/m^2^ for adults; BMI >  + 2 standard deviations (SD) over the average value given by the World Health Organization (WHO) for adolescents [55]
- Person affected by eating disorder or underweight: BMI < 18,5 kg/m^2^ for adults; BMI > -2 SD under the average value given by the WHO for adolescents [55]
- Person affected by allergy to ingredients of the lozenges
- Above-average consumption of milk products: > 3 portions/day =  > 1,2 L of milk or 1200 g yoghurt/day [56] (daily dose recommended by the German society for nutrition = 1–3 portions of milk products [56])
- Intake of antibiotics or dietary supplementation (probiotics, vitamin C/D) in the last 6 months or during the study
- Regular use of antibacterial mouth wash
- Pregnancy
- Smoking (tobacco, smokeless forms of tobacco)
- Retraction of the declaration of consent by the patient or the parents or legal guardians for adolescent patients

### Recruitment and informed consent materials

Patient recruitment takes place during regular consultation hours. To ensure that the adequate number of study participants will be enrolled and the planned sample size will be reached, the patient lists will be controlled in advance. The goal is to select patients that are suitable for recruitment, e.g., patients having an informational or control appointment before the planned insertion of a fixed appliance. After patient recruitment, the study leader (C.L.S.) as well as orthodontists, dentists, medical doctors (V.S.) and doctoral students (T.W.) working at the Department of Orthodontics and Orofacial Orthopedics at the FAU Erlangen-Nürnberg will inform patients and parents/legal guardians about the study design. Signed informed consent is mandatory prior to the beginning of the study.

Moreover, information about data protection and information material will be handed out to the patient and parents/legal guardians, including information about the study protocol, the appointments for collection of samples and measuring of clinical parameters and the investigation of clinical samples. Moreover, the benefits, risk and study cancellation criteria will be explained: Participants in the study group might have a benefit in the context of oral health since intake of probiotics was shown to reduce inflammation [45, 46]. The products of BioGaia containing *L. reuteri* were proven save in preterm babies, infants, children, healthy adults and immune compromised adults and no serious adverse effects have been observed up to the maximum tested dosage of 10 billion CFU per day, which equals 1000 times the recommended daily dose [57]. The recording of the clinical parameters, the collection of saliva samples and gingival crevicular fluid bears no risk for the study participants. Sampling of peripheral blood also poses no special risk for adults as it will be performed by licensed doctors except the usual risks of venous blood punctures, i.e. pain or hematoma/bruise at the puncture site and, in rare cases, thrombosis, local inflammation or permanent damage of blood vessels or nerves [58, 59]. Documents will be discussed with the patient and parents/legal guardians and questions will be answered (C.L.S., T.W., V.S.). Signed data protection is mandatory prior to the beginning of the study. Moreover, age, sex and gender of study participants will be noted based on a medical history sheet. The documents will be collected by the study leader and stored in a save place together with the informed consent. Besides, the nature of orthodontic treatment, malocclusion as well as the current status of occlusion will be recorded for all study participants. In case that study participants received orthodontic treatment with lose or partly fixed appliances (e.g., due to impacted canines) before recruitment, this will also be written down. The entire information will be presented as study population characteristics and used for subgroup analyses.

### Explanation for the choice of comparators

In this clinical trial, the influence of *L. reuteri* Prodentis®-lozenges (DSM 17938, ATCC PTA 5289) versus control lozenges on clinical parameters, the oral microbiota and the immune response during an orthodontic treatment in adolescent and adult patients will be compared. The active comparator *L. reuteri* Prodentis®-lozenges (DSM 17938, ATCC PTA 5289; BioGaia AB, Sweden) and the control comparator lozenges (BioGaia AB, Sweden) (Table 1) were chosen since the adjuvant use was proven beneficial concerning oral health [45, 46]. To our best knowledge, the effect of *L. reuteri* Prodentis®-lozenges, which contain two probiotic strains (DSM 17938, ATCC PTA 5289; BioGaia AB, Sweden), has not been studied during orthodontic treatment with fixed appliances so far. Since fixed appliances were shown to lead to strongest side effects shortly after insertion of appliance within the first three months [31, 32], we chose to start lozenge intake from the day of insertion of fixed appliance lasting for 12 weeks. The intake will be two times daily in accordance to the manufacturers’ instructions [60]. Lozenges consist of bulking agent (isomalt), *L. reuteri* DSM 17938 and *L. reuteri* ATCC PTA 5289, fully hydrogenated palm oil, peppermint flavor, menthol flavor, peppermint oil and sweetener (sucralose) [45, 60]. One lozenge consists of a minimum of 200 million live *L. reuteri* Prodentis [45, 60]. They are available under the name *L. reuteri* Prodentis®/™ (BioGaia AB, Sweden) [60]. Control-lozenges will be free of *L. reuteri* strains (Table 1).

**Table 1:** List of comparators

Arms	Assigned interventions
Active Comparator: *Limosilactobacillus reuteri* Prodentis®-lozenges (BioGaia)	Dietary Supplement: *Limosilactobacillus reuteri* Prodentis®-lozenges (DSM 17938, ATCC PTA 5289) Two times per day for 12 weeks
Control Comparator: Control-lozenges (BioGaia)	Dietary Supplement: Control-lozenges (BioGaia) Two times per day for 12 weeks

### Intervention description

Patients will receive lozenges and a protocol, in which they receive information about the intake and storage of lozenges:

- Supplementary intake of lozenges two times per day (1-0-1) for 12 weeks after the insertion of the fixed appliances (slowly melt on the tongue, mandatory after brushing of teeth)
- Storage of lozenges at in the refrigerator at 4 °C
- Intake protocol to control the regular intake of lozenges
- Return of canisters for counting of left overs

Further, they receive an intake protocol, in which they should make a note about the daily intake of the lozenges. To control the lozenge intake, patients will be asked to return the used canisters for counting of left overs. Further, referring to adolescent study participants, not only the study participant (adolescent) but also the parent and/or legal guardian will be asked about the maintenance of perfect storage conditions during the study time (lozenge intake). Further, a nutrition protocol will be handed out to all participants to obtain an accurate, self-reported diet history on a daily basis. Participants should list their meals for breakfast, lunch and dinner as well as snacks. Additionally, the average water consumption and drinks containing sugar should be listed. Moreover, a special focus will be on the individual intake of milk products (e.g., yoghurt, cheese, milk), which should be noted in detail. Average consumption of milk products will be allowed.

**Fig. 2:**
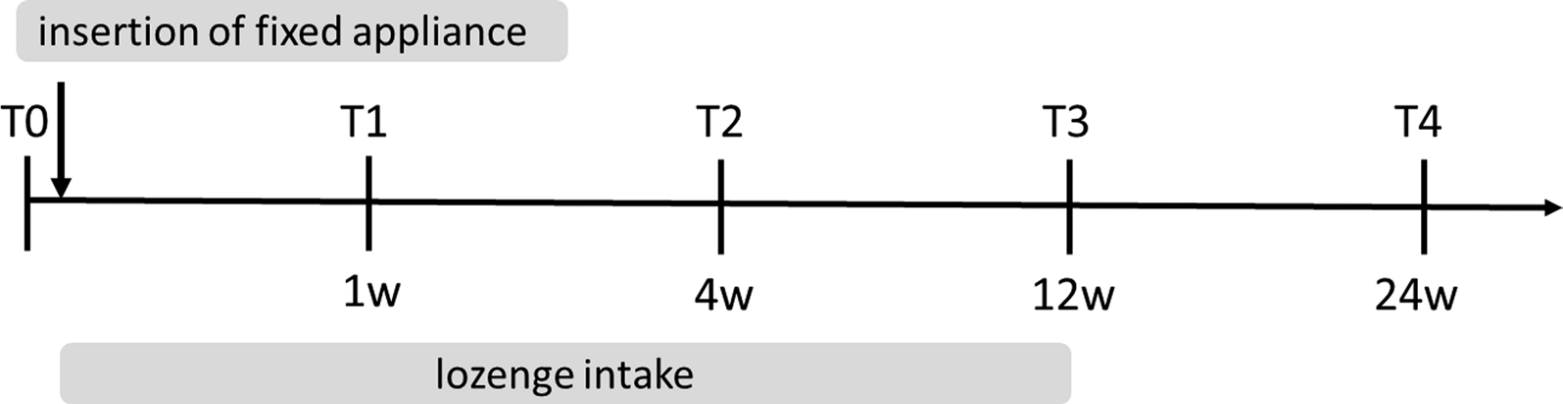
A time line showing all five study time points is given. Base line (T0) = before the insertion of the fixed appliance; T1 =  ~ one week (w) after insertion of fixed appliance/ start of lozenge intake; T2 =  ~ four w after insertion of fixed appliance/ start of lozenge intake; T3 =  ~ 12w after insertion of the fixed appliance = last day of lozenge intake; T4 =  ~ 24w after insertion of fixed appliance = 24w after start of lozenge intake = 12w after end of lozenge intake

Study participants in the test and control group will have five study time points (Fig. 2) including a base line check-up and four follow-up consultations during the study period for measurement of clinical parameters and taking of samples (Fig. 2). Sampling and examination according to the time line will be done during routine practice visits. The first consultation will be the base line check-up, where all clinical parameters and samples will be taken. Here, intake and nutrition protocol will be explained to the study participants and lozenges will be handed out to the patient. Day of insertion of fixed appliance is the start of the lozenge intake. There will be four follow-up consultations during the study period, where all clinical parameters and samples will be taken again for longitudinal evaluation. Taking of blood samples (V.S.) will only be performed on T0, T2 and T3 and only in the adult group.

### Outcomes

#### Primary outcome

##### Gingival Index

Primary endpoint is the change of the GI from baseline to week four (T0, T2). The measurement of GI is described by Löe et al*.* [61] using visual evaluation and mechanical stimulation of gingival tissues by gentle probing of the gingival sulcus, differentiation between mild, moderate and severe inflammation and scoring of the gingival condition according to defined criteria (Table 2). The scores will be measured full-mouth at four sites per tooth (the mesio-buccal, the disto-buccal, the mesio-palatinal and the disto-buccal area), added and divided by four to obtain the ‘GI for the tooth’-Index. We will use the ‘GI for the tooth’ described by Löe et al*.*, but only of ‘defined teeth’: teeth, which are without a bracket at baseline and which will be provided with a fixed bracket at the day of insertion of the fixed appliance. In case of fixed full appliance in both arches, the GI will be measured full-mouth considering both arches, however, in case of fixed appliance only in the lower or the upper arch, the GI will only be measured full arch in the arch receiving orthodontic treatment. The ‘GI for the patient` is then the mean of the GIs for the ‘defined teeth’. Moreover, for longitudinal investigation of gingival inflammation, the GI will also be measured longitudinally till six months after insertion of the fixed appliance (T1, T3, T4).

**Table 2:** Criteria for the Gingival Index described by Löe et al. [61]

Score	Gingival condition	Description
0	Absence of inflammation	No clinical sign of inflammation
1	Mild inflammation	Slight change in color and little change in texture, no bleeding on probing
2	Moderate inflammation	Moderate glazing, redness, edema, hypertrophy, bleeding on probing
3	Severe inflammation	Marked redness and hypertrophy, tendency to spontaneous bleeding, ulceration, edema

### Secondary outcome variables

#### Periodontal probing depth (PPD)

For the investigation of gingival and periodontal status, the PPD will be measured longitudinally from baseline till six months after insertion of the fixed appliance (T0, T1, T2, T3, T4). The measurement of the PPD is performed according to standardized protocols [62, 63]: insertion of a WHO periodontal probe (probe tip with a diameter of 0.50 mm) into the gingival sulcus with a force of 0.2–0.3 N [64, 65]; probing depth is read out at landmarks on the WHO periodontal probe [62, 63]. The scores will be measured full-mouth at four sites per tooth, added and divided by four to obtain the ‘PPD for the tooth’-Index. We will use the ‘PPD for the tooth’, but only for ‘defined teeth’ (explained above for GI). The ‘PPD for the patient’ is then the mean of the PPDs of the ‘defined teeth’.

### Modified Plaque Index (MPI) by Attin et al*.* [66]

For the evaluation of oral hygiene, the MPI will be measured longitudinally from baseline till 6 months after insertion of the fixed appliance (T0, T1, T2, T3, T4). The measurement of MPI is described by Attin et al*.* [66], which scores the amount of plaque according to the defined criteria (Table 3). The score will be measured full-mouth at each tooth, but only for ‘defined teeth’ (explained above for GI). The ‘Modified plaque index for the patient ‘ is then the mean of the MPIs of the ‘defined teeth’ (Table 2): Index = (Sum of MPI per tooth × 100)/(3 × number of measured teeth). To measure MPI, we will use the following disclosing agent: Mira-2-Ton®, a two-tone erythrosine-free disclosing dye solution (Hager & Werken, Duisburg, Germany), which can help to differentiate blue-dyed elderly plaque from pink-dyed recent plaque. The MPI will be determined clinically by direct assessment of the study participant.

**Table 3:** Criteria for the Modified Plaque Index (MPI)

Score	Oral hygiene	Description
0	Very good	No plaque
1	Good	Small plaque areas approximal
2	Moderate	Small plaque areas approximal + cervical
3	Poor	Plaque covers 1/3 of the cervical area of the bracket

### Local cytokine concentration

Sample collection: For the analyses of local inflammation, samples from three different oral niches will be collected longitudinally from baseline until 6 months after insertion of the fixed appliance (T0, T1, T2, T3, T4). Oral niches were selected as one representative of the previously defined three metaniches [52]: 1. Tongue (representing S-T-HP), 2. cheek (representing C-U), 3. gingival crevicular fluid (representing P-GCF) [52]. Study participants will be advised not to eat or drink anything except water up to three hours prior to the study appointment. Therefore, smear soft tissue samples will be collected in Eppendorf tubes from the middle part of the tongue and molar region on the right side of the cheek using sterile swabs wiping over the area several times for about 30 s [52]. Moreover, samples of GCF will be collected from 6 periodontal pockets of the mesio-buccal side of’defined Ramfjord teeth’: 1) in case of fixed appliance in the upper and lower arch ‘defined Ramfjord teeth’ are 16, 21, 24, 36, 41 and 44; 2) in case of fixed appliance in the upper arch only ‘defined Ramfjord teeth’ are 16, 21, 24 and their mirrored partners 26, 11, 14 and 3) in case of fixed appliance in the lower arch only ‘defined Ramfjord teeth’ are 36, 41, 44 and their mirrored partners 46, 31, 34. In case of a missing Ramfjord tooth [63, 64] we will collect GCF from the adjacent and comparable tooth (incisor, premolar, molar). GCF will be gathered using sterile paper strips (Oraflow Inc., New York, USA) in accordance to standardized protocols [21, 22, 52, 67, 68]: usage of cotton rolls for draining, smooth air-drying of the ‘defined Ramfjord teeth’ and careful insertion of paper strips in the gingival sulcus (premolar/molar region 30 s, incisor region 60 s). Sulcus fluid flow rate (SFFR) will be measured using Periotron 8000 (Oraflow Inc., New York, United States) according to the manufacturer’s instructions and will be used to determine the volumes of gingival crevicular fluid according to a calibration curve in order to calculate original cytokine concentrations [52]. Samples will be frozen immediately after collection according to standardized protocols [52].

Evaluation of local cytokine concentration: Tongue and cheek samples will be eluted from swabs by centrifugation and dilution to > 100 µl (Meso Scale Discovery, Rockville, USA, R50AG-2). Elution of proteins samples of GCF collected with six paper strips of one patient will be performed in cold diluent buffer (Meso Scale Discovery, Rockville, USA, R50AG-2). The concentration (in pg/mL) of GM-CSF, IFN-γ, IL-2, IL-4, IL-6, IL-8 and IL-10 as well as TNF will be measured using multiplex immunoassay with a U-PLEX Biomarker Group 1 (hu) assay (Meso Scale Discovery; Rockville, USA, K15067L-2) on a MESO QuickPlex SQ 120 instrument (Meso Scale Discovery; Rockville, USA) in accordance with the manufacturer’s instructions, standardized dilution sequences and in accordance with previous studies [25, 52].

### Oral microbiota

Sample collection: For the investigation of the composition of oral microbiota, samples will be collected in accordance to the sample collection method described above for investigation of local cytokine concentrations using standardized protocols [21, 22, 52, 67, 68] and ‘defined Ramfjord teeth’ are the same as described for local cytokines.

Microbial DNA will be isolated with the ZymoBIOMICS DNA Microprep kit according to manufacturer’s instructions (Zymo Research, Freiburg, Germany). Composition of the oral microbiota will be analysed using 2 × 300 bp paired-end sequencing of the 16S rDNA V1-V3 regions on a MiSeq system (Illumina, San Diego, CA, USA) in accordance with previous studies [52]. Raw reads will be subjected to quality-based filtering and primer sequences will be removed using a combination of Trimmomatic [69] and Cutadapt [70]. Subsequently, we will perform classification to operational taxonomic units (OTUs) and identification of zero-radius OTUs (ZOTU) based on the USEARCH 11 algorithm [71, 72] as implemented in the IMNGS web server [73] and taxonomic classification using the SILVA databases will be used [52, 74]. Further analyses with regard to alpha and beta diversities, correlation and enrichment of OTUs or taxa will be done within R [75] vegan [76] and edgeR [77].

### Serum cytokine levels

Sample collection: For the investigation of systemic inflammation, samples of peripheral whole blood (WB) will be taken by puncturing a peripheral (e.g., cubital or cephalic) vein in accordance to standardized protocols [58]: The patient is positioned upright in an adjustable chair followed by application of a tourniquet, selection of a puncture site on the non-dominant arm, site disinfection with a local antiseptic solution (Aseptoderm, Dr. Schumacher, Malsfeld, Germany), careful insertion of a butterfly-needle (Safety-Multifly® 21G tube200mm, Sarstedt AG, Nümbrecht, Germany) and taking of blood samples with vacuum syringes with heparine coating(50S-Monovette, Sarstedt AG, Nümbrecht, Germany) [58]. The congestion-to-sample time is kept below 60 s to avoid changes in the blood sample and, after sample collection, the tourniquet and the needle are removed and the puncture site is compressed for 2 min with a sterile compress, followed by adequate dressing [58]. WB samples are taken before insertion of the fixed appliance (T0; baseline), 4 weeks after insertion of fixed appliance and after start of lozenge intake (T2) and 12 weeks after insertion of fixed appliance at the end of lozenge intake (T3) (Fig. 2).

Serum cytokine analyses: Serum will be separated from peripheral whole blood by centrifugation at 2000×*g* for 10 min and stored at -80 °C. Measurement of systemic cytokine concentrations in serum (in pg/mL) will be performed using enzyme-linked immunosorbent assays (ELISA; e.g., Human IL-17 ELISA kit (ab100556), Abcam, Cambridge, UK) or multiplex immunoassay (U-PLEX Biomarker Group 1 (hu) assays from Meso Scale Discovery, Rockville, USA) according to the manufacturer’s instructions and in accordance to previous studies [78–81]. To analyse systemic inflammation and a possible Th_1_ cell (Th_1_) /Th_2_ cell (Th_2_) or Th_17_ shift, pro-inflammatory cytokines and cytokines representing the Th_1_/Th_2_/Th_17_ cytokine profile (e.g. TNF, IFN-γ, IL-10/-17) will be measured [82].

### Frequencies of innate and adaptive immune cells

#### Sample collection

For the investigation of cellular immunity, samples of (heparinized) peripheral whole blood (WB) will be taken by puncturing a peripheral (e.g., cubital or cephalic) vein in accordance with standardized protocols as explained above [58] before insertion of fixed appliance (T0; baseline), 4 weeks after insertion of fixed appliance and after start of lozenge intake (T2) and 12 weeks after insertion of fixed appliance at the end of lozenge intake (T3) (Fig. 2).

Analyses of frequencies of innate and adaptive immune cells: Peripheral blood mononuclear cells (PBMNC) will be separated from whole blood samples by density gradient centrifugation using the Ficoll-Hypaque technique (Biocoll separating solution; Biochrom, Berlin, Germany), washed, suspended in phosphate-buffered saline (PBS; Biochrom, Berlin, Germany) and frozen according to standardized protocols [81, 83–85]. The cellular immune status will be analysed quantitatively and qualitatively (frequencies of immune cells and their subtypes in the PBMNC-fraction) with thawed PBMNC using multiparametric flow cytometry (Cytoflex S, Beckman Coulter, Indianapolis, United States) [81, 83–85]. Lymphocyte typing will be performed using an ‘inflammatory panel’ to quantify different innate and adaptive immune cells, e.g., different CD4 or CD8 T cell subtypes (activated, proliferating/non-naïve/memory (T_pro_, T_non-naïve,_ T_mem_), T helper cells (Th_1_/Th_2_/Th_17_ cells) or regulatory T cells (T_regs_), B cells, natural killer (NK) cells, natural killer T (iNKT) cells and dendritic cells (DC) [81, 83, 84].

### Participant timeline

Time schedule of enrolment, interventions (including any run-ins and washouts), assessments, and visits for participants is depicted in Table 4.

**Table 4:** Time schedule of enrolment, interventions and assessments according to the SPIRIT statement

	Enrolment	Allocation	Post-allocation					Close-out
TIMEPOINT	-T_0_	0	T_0_	1w	4w	12w	24w	24 weeks
ENROLMENT:								
Eligibility screen	X	X						
Informed consent	X							
*Data protection, Information material*	X							
Allocation		X						
INTERVENTIONS:								
*L. reuteri* Prodentis®-lozenges (DSM 17938, ATCC PTA 5289)								
Control lozenges								
ASSESSMENTS (NCT04598633* and NCT04606186*):								
Gingival Index			X	X	X	X	X	X
Probing Pocket Depth			X	X	X	X	X	X
Modified Plaque Index			X	X	X	X	X	X
Local cytokine concentration^1^			X	X	X	X	X	X
Oral microbiota^1^			X	X	X	X	X	X
ASSESSMENTS (NCT04606186*):								
Serum cytokine levels ^2^			X		X	X		
Frequencies of innate and adaptive immune cells^2^			X		X	X		

*Clinical Trial Registration number (registered at ClinicalTrials.gov in two parts: NCT04598633 for adolescents and NCT04606186 for adults); ^1^ samples from three oral niches: tongue, cheek, gingival crevicular fluid; ^2^ peripheral blood samples

### Sample size

The primary endpoint is the change of the Gingival Index from baseline to week four. The selected studies [45, 46, 86–88] were designed as randomized controlled trials (RCTs) and compared *L. reuteri* lozenges with control in various populations (Table 5). Effect size was calculated as follows: the change of the GI at a follow up visit and baseline was calculated. Then, the difference of this change in treatment and the control group was calculated and divided by the pooled standard deviation. Effect size varies across the studies. Notably, Iniesta et al*.* [88] reported no effect of lozenges compared to control. The other studies reported a significant effect [45, 46, 86, 87]. The smallest effect size of 1.00 was reported by Schlagenhauf et al*.* 2016 [45]. We use this worst case for the sample size estimation.

**Table 5:** Effect size of *L. reuteri* lozenges versus control lozenges on Gingival Index (GI)

Paper	Effect size	Number of study participants
Schlagenhauf et al*.* [45]	1,00	55
Schlagenhauf et al*.* [46]	1,33	72
Vivekananda et al*.* [86]	2,79	30
Tekce et al*.* [87]	1,52	40
Iniesta et al*.* [88]	0,00	40

It cannot be assumed that the change in GI is normally distributed. Hence a Wilcoxon rank-sum test (WRT) is used to decide for significance. As the WRT is non-parametric, a sample size estimation for this test is not possible. Blair et al*.* 1980 [89] compared WRT with the t-test. The comparison showed that the power of the WRT is comparable to the power of t-test for various distributions. In the worst case, the WRT showed a power that was 5 percentage points lower than the power of the t-test. Thus, we will perform a power calculation for the t-test with 85% power and get the power of WRT of at least 80%.

Sample size estimation is performed with the following parameters: Two sample t-test, alpha = 0.05, Power = 0.85, one-sided test (superiority), H0: no difference between treatment and control and H1: difference in means between treatment and control is at least one SD (effect size > 1). The sample size calculation yielded as a result 16 study participants per arm, i.e. 32 participants in total. A greater effect size will yield smaller sample sizes. Sample size was calculated with Software R [90] using the function’power.t.test’ by the CCS (A.S.). The formula for this sample size estimation is n = (σ/δ)^2^ * (z_1−α_ + z_1−β_)^2^ where δ/σ is the effect size and z_1−α_ is the 1-α-quantile of the t-distribution. We estimate that two study participants will drop out till week four. This corresponds to a low drop-out rate of 5%, in line with the selected studies [45, 46, 86–88]. Therefore, 34 patients have to be recruited. The analysis of the primary endpoint is applied to the per-protocol population. Therefore, the number of cases will be 17 study participants for each study and each control group: 34 adolescents (17 study participants for the study group, 17 study participants for the control group) and 34 adults (17 study participants for the study group, 17 study participants for the control group).

### Sequence generation

The algorithm for the creation of the randomization list is the Maximal Procedure [53]. The Maximal Procedure is implemented in the R Package randomizeR [91]. Parameter for the Maximal Procedure are: Number of patients 34, Maximum Tolerated Imbalance (MTI) is set to 4 and Allocation ratio is 1:1.

This randomization is conducted twice: for adults and adolescents.

### Concealment mechanism

The randomization list for both groups generated by an external person working at the Center for Clinical Studies (CCS) of the University Hospital Erlangen is arranged into single tickets for each pseudonym for each study participant in the continuing order. The printed tickets are then put into opaque envelopes labelled with a consecutive randomization number. Then, the envelopes are taken to the Department of Orthodontics and Orofacial Orthopedics at the FAU Erlangen-Nürnberg and handed out to the lab manager. The study design is double-blind. Therefore, neither the clinical examiners nor the study participants will have knowledge about the randomization plan.

### Implementation

The company BioGaia will provide 6 closed storage boxes, containing 30 lozenges, for each of the 68 study participants in advance. Half of the canisters will contain test-lozenges (study group), the other half will be filled with control-lozenges (control group). Canisters are stored in a refrigerator in a save and lockable room at the Department of Orthodontics and Orofacial Orthopedics at FAU Erlangen-Nürnberg. Before the first appointment of an included study participant, the lab manager opens the envelope containing the assignment of the study participant to test or control group. After this process, the storage boxes will be labelled by the lab manager with the pseudonym of the study participant in accordance to the assignment in the envelope and the randomization plan generated by the CCS. The lab manager will hand over the storage canisters to the study leader before the first appointment, who will store them in a refrigerator. Each study participant will receive 6 closed storage canisters, containing 30 lozenges each, labelled with the pseudonym at the end of the regular consultation to ensure short transport times from the department to the private refrigerator in the home of the study participant. Therefore, study participants will receive five canisters. Half of them will receive lozenges containing probiotics, half of them will receive control-lozenges.

### Who will be blinded and procedure for unblinding if needed

The study design is double blind. The storage boxes for the test and the control group will be identical and blinded. The study participants and examined raters will be blinded. Moreover, the whole clinic personnel will not have knowledge of the group affiliation of study participants. The only person having knowledge about the group affiliation will be the lab manager.

If unblinding is necessary due to any circumstances, e.g., the occurrence of AE, the lab manager will be informed to reveal the study participants allocation to the test or control group.

### Criteria for discontinuing or modifying allocated interventions

The exclusion criteria will not only be checked using a medical history sheet initially, but also measured and controlled regularly during the study period. If the study participant did not meet the exclusion criteria at base line, but does during the course of the study, the study participation will be canceled. All time points of analyses will be performed during routine practice visits to ensure adherence to the clinical trial. Therefore, slight variations of study time points will not lead to exclusion or discontinuation of the study. However, the exact study time point will be analysed as SD of the mean study time point and evaluated in the analyses and interpretation of the results. If the study participant decides to stop intake of lozenges due to any reason, the study participation will be terminated. The participation in the study is voluntary and a refusal to participate will not lead to disadvantages or changes in the orthodontic treatment. The withdrawal and cancellation of the study will be possible at any time during the study and will not lead to disadvantage or influence of the orthodontic treatment.

### Strategies to improve adherence to interventions and plans to promote participant retention and complete follow-up

All time points regarding the study procedure will be matched with routine practice visits. Thus, extra effort of the study participants can be minimized and the adherence to the clinical trial can be ensured. Study participants will receive a painted colorful intake and nutrition protocol as a motivational reminder, e.g., hung up on the refrigerator, in which the lozenges are stored. Since adolescents might not participate in the study, if taking of blood samples is mandatory, the taking of blood samples will only be performed in the adult group on 3 appointments. Adults with fear of needles, who would like to participate in the trial, will be enabled to participate without taking of blood samples (study design is then the same as for adolescents). If an adult seeks to participate in the study but is afraid of needles and/or injections, he will also be enabled to be part of the study. The aim of these strategies is to assure the achievement of the planned sample size within the scheduled time frame. Since orthodontic treatment with fixed appliances usually lasts for 18 to 24 months, all study participants will continue to show up to regular consultations in the Department of Orthodontics and Orofacial Orthopedics. Hence, a follow-up of study participants is given.

### Relevant concomitant care permitted or prohibited during the trial

Study participants do not receive any concomitant care permissions or prohibitions during the trial.

### Provisions for post-trial care

Study participants do not receive post-trial provisions. Since all study participants continue to have regular consultations in the department due to their ongoing orthodontic treatment with fixed appliances, which usually last 18 to 24 months, a follow-up of study participants is guarenteed.

### Plans for assessment and collection of outcomes

The clinical data assessment for this trial was standardized and trained in advance in a recent clinical study [52]. Collection of samples and clinical parameters will be performed by examined raters (C.L.S., T.W., V.S.), who were trained in preliminary experiments to guarantee inter- and intra-examiner accuracy and a high data quality. Clinical parameters including the GI, PPD, MPI as well as the sulcus fluid flow rate (SFFR), which is measured during the appointment using the Periotron device, will be noted chair-side on a data sheet, which was designed in a previous study [52] and modified for this trial in advance. Immediately after collection of the clinical data on the data sheet, the data are transferred to a Microsoft Excel spreadsheet and stored in a secure folder with restricted access only to study staff.

### Data management and confidentiality

All participants of the study receive pseudonyms saved in a special program with restricted access only to the leader of the study (C.L.S.). Patient data and collected data are processed according to the Guidelines of Data Protection set by the ‘Universitätsklinikum Erlangen and Friedrich-Alexander-Universität (FAU) Erlangen-Nürnberg' (Art. 6 Abs.2 Nr. 3 c BayDSG; data protection commissioner: Jan Köster; jan.koester@uk-erlangen.de).

### Plans for collection, laboratory evaluation and storage of biological specimens for genetic or molecular analysis in this trial/future use

After analyses, all samples will be disposed and destroyed properly.

### Statistical methods

Descriptive analysis is applied according to the scale of measurement of the variable: Nominal and ordinal variables are described by counts and percentages. Interval scaled variables are described by mean and standard deviation, or robust estimators like quantiles if necessary. The number of missing and valid values are counted for every variable. Inference statistics is based on a frequentist basis with hypothesis tests, p-values and confidence intervals. Applicable tests for this two-arm study are the t-test, WRT and Chi^2^-tests, according to the scale of measurement of the variable. The significance level is set to 5%; confidence intervals are two-sided with a length of 95%. Corrections for multiple testing may be applied, when a hypothesis is tested in multiple subgroups. The protocol presented in the paper is according to the SPIRIT guidelines [92, 93]. The size of the populations is reported by the Safety Evaluation Set (SES), Full analysis Set (FAS) and the Per Protocol Set (PPS). If these populations have remarkably different size, all endpoints and demographics are reported for each population. The software R will be used for data analysis. The following analysis sets will be defined for the statistical analysis of this study: (1) Safety Evaluation Set: The SES is the subset of all subjects who were exposed to any study intervention at least once; (2) Full Analysis Set: The FAS is the subset of subjects in the SES for whom the primary endpoint is available (here: all subjects who have GI data available at baseline and at four weeks); (3) Per Protocol Set: The PPS is the subset of subjects in the FAS without major protocol deviations. One major protocol deviation is the noncompliant intake of lozenge. Further major protocol deviations may be defined during the study.

An Interim analysis is not planned. Additional analyses will be performed to compare adolescents with adults. Subgroup analyses will be performed to compare study participants with inserted fixed full appliance in upper and lower arch versus inserted fixed full appliance in upper arch versus inserted fixed full appliance in lower arch. In case of missing data, e.g., the study participants missed one of the four follow-up consultations (T1-T4), the time point will not be evaluated for the study participant. For example, if the study participant misses T1, but show up to T2, the evaluation of the primary endpoint is possible for the study participant, however, longitudinal analyses is only possible after T2. Data will not be shared.

### Oversight and monitoring, composition of the coordinating centre and trial steering committee

Not applicable.

### Adverse event reporting and harms

The study participants will be advised to report any AE during the study period immediately and will be explicitly asked for the occurrence of AE during the study period on every time point of the collection of samples. In case of AE, the study participation will be canceled and intake of lozenges will be stopped immediately. Since adolescent patients are included in this trial, not only the study participant (adolescent) but also the parent and/or legal guardian will be asked about the occurrence of AE. Corrosion of orthodontic archwires can occur due to saliva (composed of inorganic salts, organic and gastric acids) and/or biofilm properties (community of microorganisms attached to the surface of teeth and archwires) [94]. Studies analysed the effect of probiotics on corrosion of different types of archwires and orthodontic materials: nickel-titanium (NiTi) alloys (uncoated, nitrified and rhodium coated surface) [94, 95], titanium and stainless steel (SS) [94, 96]. A mixture of artificial saliva with dissolved *L. reuteri* Prodentis® DSM 17938 and ATCC PTA 5289 (BioGaia, BioGaia AB, Sweden) was shown to have less corrosive effects on an uncoated surface (BioForce Sentalloy®, Dentsply International GAC, United States), comparable effects on nitrified surfaces and higher local and general corrosive side effects on rhodium coated surfaces compared to artificial saliva alone, indicating that surface roughness induced by probiotic was not greater than induced by saliva [95]. Contrary, Trolic et al*.* demonstrated that a probiotic-saliva mixture increased the surface roughness in uncoated NiTi, but decreased roughness of rhodium or titanium nitride coated NiTi and SS archwires [94]. Pavlic et al*.* concluded that after insertion of orthodontic mini-implants, *L. reuteri* can reduce corrosion of SS mini-implants, while chlorhexidine should be favoured for titanium implants [96]. Regarding safety of probiotics during orthodontic treatment with fixed appliances, probiotics appear to decrease corrosion of SS archwires [94, 96] and to influence corrosion of NiTi archwires at least to some extent, depending on the surface coating; however, different studies yielded contradictory results [94, 95]. NiTi archwires with uncoated surface (BioForce Sentalloy®, Dentsply International GAC, United States) are standard archwires used at the beginning of the orthodontic treatment in the Department of Orthodontics and Orofacial Orthopedics, while stainless steel archwires (3 M™ Unitek™, United States) are standard archwires used in advanced treatment with fixed braces. Standard brackets used for orthodontic treatment with fixed appliances in the Department for Orthodontics and Orofacial Orthopedics are metal brackets (Sprint ® II and Mini-Sprint ® II) with MBT 5.0 prescription (Forestadent, Pforzheim, Germany) [97]. Hence, even in case of any corrosive influence on archwires due to *L. reuteri* Prodentis®, surface roughness and potential influences on the oral microbiome and local inflammation will be similar in the study and control group due to standard archwire sequences. Further, all used orthodontic archwires and type of brackets will be reported as characteristics of the study population.

### Plans for seeking research ethics committee/institutional review board (REC/IRB) approval

The study protocol and written documents for the study participants (declaration of consent, data protection, information material) were reviewed by the local ethics committee and requested modifications were included. The study protocol and written documents were approved by the local ethics committee of the Friedrich-Alexander Universität (FAU) Erlangen-Nürnberg (Krankenhausstraße 12, 91054 Erlangen, Vote number: 347_19B, Vote Date: 15/10/2019). The local ethics committee will be seeked in case of important protocol amendments. Important protocol amendments will be reported directly to the local ethics committee of the Friedrich-Alexander Universität (FAU) Erlangen-Nürnberg (Krankenhausstraße 12, 91054 Erlangen, Vote number: 347_19B) and to the company BioGaia.

### Access to data and dissemination plans

Once the clinical part of the trial has been accomplished and all data have been collected, data will be evaluated and interpreted by principal investigator, who has access to all data, and co-investigators, who will have access to their own site’s data sets. The principle investigator as well as the co-investigators are going to publish the findings in international peer-reviewed journals. The use of professional writers is not intended. The aim is to publish in journals, which are open access so that the information is widely accessible. Participants of the study will be informed about the findings of the trial, but only if desired. Prior to submission of any publication or disclosure, the study leaders will provide BioGaia with drafts of the proposed publications and disclosures, whether oral or in writing. BioGaia shall request a delay in publication for a period of eight weeks in order to allow patent applications or to remove confidential information.

## Discussion

Orthodontic treatment with fixed appliances leads to an increase of plaque niches, biofilm accumulation and reduction of self-cleaning [5] and thereby might induce gingival inflammation and increase the risk for periodontal destruction [3]. The aim of this study is to evaluate the use of probiotics as a possible preventive treatment approach during orthodontic treatment with fixed appliances. Probiotics were shown to improve clinical parameters in periodontal therapy [39]. However, so far no positive effects were seen with respect to WSL [48], gingival indices and BOP during orthodontic treatment [49–51], which was explained by several risks of bias [47]: (i) The intake of lozenges started only late during treatment, at a time when alterations in oral health (e.g., oral inflammation, higher amounts of plaque, changes in the oral microbiota and local or systemic immune responses) might have already occurred. (ii) Lozenge intake was instructed no longer than four weeks [47], while trials investigating the use of lozenges in periodontal therapy chose longer intake periods (7 weeks for study participants with pregnancy gingivitis, 42 days for navy sailors at sea) to ensure an adequate time for oral health to recover [45, 46]. Furthermore, it was indicated that the use of more than one probiotic strain might be more efficacious [47]. (iii) Trials did not analyse several longitudinal short-term appointments during the intake of lozenges to investigate how long it takes until changes of oral health are detectable. (iv) Follow-up appointments after intake of lozenges were lacking to analyse whether a potential beneficial effect of probiotics was solely seen in the short term or was long-lasting. (v) The studies only analysed one or two parameters, e.g. GI and PI, but did not investigate other important factors representing oral health like the complexity of the oral microbiome. In the current study, we therefore chose a comparator which has proven to be beneficial in patients at risk for gingivitis and periodontal disease [45, 46] and contains two probiotic strains: *L. reuteri* DSM 17938, *L. reuteri* ATCC PTA 5289 [60]. Moreover, we will analyse intake of lozenges for a longer time period (12 weeks) to allow longer recovery and we will start intake at the day of insertion of fixed appliances to avoid the flare-up of inflammation prior to lozenge intake. Finally, we have implemented several longitudinal study time points before insertion of fixed appliances/lozenge intake and during lozenge intake and will have a follow-up appointment 3 months after lozenge intake.

Regarding clinical parameters, we chose the GI as a primary outcome objective, since (i) it was used in studies presenting beneficial effects of probiotics [45, 46, 86–88], (ii) it was considered the most common index used in preventive/ therapeutic clinical trials evaluating gingival inflammation [98], (iii) it has been supported by histological studies (high GI: infiltrated connective tissue) [99] and (iv) it provides more parameters than other gingival indices, e.g., BOP-Index solely determines the presence of bleeding [100] and the modified gingival index (MGI) uses only visual parameters [101], which is biased due to subjective measurements of color changes of the gingiva [98]. Regarding plaque accumulation, we will use the modified plaque index (MPI) [66], since it allows to describe the amount of plaque not only in approximal areas like the approximal plaque index (API) [61], but also on the cervical area and around the bracket since additional plaque retention niches need to be evaluated in orthodontic patients and since probiotics reduced plaque accumulation [45]. To enable the reevaluation of the index and to ensure reproducibility, intraoral photographs [102, 103] will be taken after staining of teeth. Since our study will focus on effects of fixed brackets, the GI [61], MPI [66] and PPD will be measured full-arch but only on teeth receiving fixed bonded orthodontic brackets.

Regarding oral microbiota, it was reported [31, 32] that well-known key pathogens of the red complex [104] increased 3 (to 6) months after the insertion of fixed braces, but returned to initial levels 6 months after insertion [32], indicating that preventive treatment strategies should aim to reduce or inhibit an increase of pathogenic species directly after insertion within the first three months. Since the intake of probiotics [105] was shown to decrease key pathogens [104], to change the composition of the oral microbiome [106] and to reduce leukotoxin expression [107], we chose probiotics as a preventive treatment approach for three months after insertion and a study period of 6 months after insertion of appliance. A longer observation time might be interesting, however, as the cooperation of the adolescent study participants is required, we chose the proposed observation time [31]. The complex theory [104] was displaced by an updated theory defining periodontal health as a symbiotic microbial community with a balance between protective, aerobic, gram-positive and anaerobic, pathogenic species and periodontitis as an inflammatory dysbiotic disease, where the relative increase of pathogenic versus protective bacterial species can lead to inflammation with an increased expression of pro-inflammatory cytokines [7]. Further, the knowledge about the pathogenic and protective bacterial species has increased: (i) greater diversity and abundance of bacterial species is found in periodontal disease [108–110], (i) lower diversity in periodontal health [110], (iii) the greater diversity was found in metaniche P-GCF compared to metaniche S-T-HP and C-U [52], (iv) age-related changes of oral microbiota with higher alpha diversity were observed in adults [35, 36, 111] and (v) males were shown to have higher prevalence for periodontal disease [112–114]. Therefore, we decided to investigate adolescents and adults separately, to analyze systemic effects only in adults and to analyse the influence of sex and gender in subgroup analysis. Further, it is still unknown which interaction of pathogens and locally changing factors leads to dysbiosis [111], which is problematic in orthodontic treatment, since a flare-up of periodontal inflammation increases the risk for tooth and bone bone loss [115, 116] and root resorptions [28]. Since one theory regarding the mechanism of orally applied probiotics is that they have direct effects on dental plaque by resistance to colonization [117, 118], we will analyze the composition of the oral microbiota to evaluate possible changes when applying probiotic strains orally.

Orthodontic treatment with fixed appliances induces an aseptic pseudo-inflammatory response [19, 20]. An increase of pro-inflammatory cytokines correlates with periodontal destruction [22–27] and orthodontic treatment can reinforce dysbiotic inflammatory periodontal disease with IL-6 playing a key role [119]. Probiotics seem to induce immunomodulatory effects by (i) strengthening the innate immune response, (ii) promoting the production of anti-inflammatory cytokines, and by (iii) inhibiting pro-inflammatory cytokines [40–44]^117^,^105^, ^120^. However, to date there are no studies that analyzed the local cytokine concentration in different oral niches [52] longitudinally after insertion of fixed appliances.

Considering systemic effects, several studies [7] presented an influence of periodontal disease on systemic parameters [121], e.g., increased numbers of different T cell subsets [122–124], B cell subsets [122],Th_17_ and Th_1_ cells [125], NK cells [126]^122^ and dendritic cells [127]. Even in periodontally healthy patients, systemic inflammation can be observed, e.g., in patients with greater mean periodontal pocket depth, gingivitis and higher levels of plaque [128]. Notably, insertion of orthodontic archwire was shown to increase systemic reactive oxygen species formation and the ensuing antioxidative defense [129]. These findings indicate that in our study population systemic effects might be seen after insertion of fixed appliances, since insertion of fixed appliances was shown to induce gingivitis, enhance plaque levels and periodontal pocket depth [3, 5]. With regard to the protective mechanisms of probiotics, indirect effects via modulation of the innate and adaptive immune system have been described [117, 118], e.g., reduction of bacterial cytotoxicity and cytokine release by PBMNCs [130], regulation of DC activity, NK-DC crosstalk and T-cell polarization [82, 131, 132], growth reduction of oral squamous cell carcinoma stem cells [133], improvement of pre-seasonal sublingual immunotherapy efficiency [134] as well as anti-allergic effects [135]. Therefore, it will be elucidated whether intake of *L. reuteri* Prodentis®-lozenges correlates with less systemic inflammation in adults.

### Trial status

The study protocol has been approved by the local ethics committee and registered at ClinicalTrials.gov in 2 parts prior to the beginning of the trial. After written informed consent and fulfilment of the inclusion criteria, study participants were enrolled in the study. Up to now, 46 study participants (15 adults [sex: 7 females, 8 males], 28 adolescents [sex: 14 females, 14 males]) have been recruited, screened for eligibility and allocated. Post-allocation study period started for 40 study participants (13 adults, 23 adolescents) in accordance with the Helsinki protocol. No data have been analysed so far. At the present phase of the trial, we are actively recruiting the remaining 22 study participants (19 adults, 6 adolescents).

## Data Availability

The principal investigator has access to all data and co-investigators have access to their own site’s data sets. Participants of the study will be informed about the findings of the trial, but only if desired. Data will not be shared with respect to access to the full protocol, participant level-data and statistical code. Raw sequencing data together with non-personal metadata will be made available through a publicly accessible database. Upon reasonable request and after approval by data protection commissioner, further de-identified metadata may be made available by the corresponding author once the trial is completed.

## Abbreviations

*A*.: *Aggregatibacter*
AE: Adverse events
API: Approximal Plaque Index
BMI: Body Mass Index
BOP: Bleeding on Probing
C: Cheek
CdtB: Cytolethal distending toxin
CRP: C-reactive protein
DC: Dendritic cell
DPP: Dentine phosphoprotein
ELISA: Enzyme-linked immunosorbent assay
FAS: Full Analysis Set
*F.*: *Fusobacterium*
GCF: Gingival crevicular fluid
GI: Gingival Index
GM-CSF: Granulocyte–macrophage colony-stimulating factor
HP: Hard palate
IFN-γ: Interferon gamma
IL: Interleukin
IOTN: Index of Orthodontic Treatment Need
*L.*: *Limosilactobacillus (reuteri)*
*L.*: *Lactobacillus (gasseri/salivarius/paracasei/* *rhamnosus* *)*
MNC: Mononuclear cell
MOP: Modified Orthodontic Plaque Index
MPI: Modified Plaque Index
MTI: Maximum Tolerated Imbalance
NK cell: Natural killer cell
NKT cell: Natural killer T cell
OHRQOL: Oral health-related quality of life
OIIRR: Orthodontically induced inflammatory root resorptions
OTU: Operational taxonomic unit
P: Plaque
PBMNC: Peripheral blood mononuclear cells
*P*.: *Porphyromonas (gingivalis)*
*P*.: *Prevotella (intermedia)*
PI: Plaque Index
PPD: Periodontal probing depth
PPS: Per Protocol Set
RCT: Randomized controlled trial
S: Saliva
*S.*: *Streptococcus*
SD: Standard deviations
SES: Safety Evaluation Set
SFFR: Sulcus fluid flow rate
T: Tongue
*T.*: *Tannerella*
